# Estrogen receptors genotypes and canine mammary neoplasia

**DOI:** 10.1186/s12917-019-2062-y

**Published:** 2019-09-10

**Authors:** Ana Canadas-Sousa, Marta Santos, Bárbara Leal, Rui Medeiros, Patrícia Dias-Pereira

**Affiliations:** 1 Department of Pathology and Molecular Immunology, Instituto Ciências Biomédicas Abel Salazar, ICBAS – UPorto, University of Porto, Porto, Portugal; 2 Department of Microscopy, Instituto Ciências Biomédicas Abel Salazar, ICBAS – UPorto, University of Porto, Porto, Portugal; 3 Molecular Oncology and Viral Pathology Group, IPO-Porto Research Center (CI-IPOP), Portuguese Oncology Institute of Porto (IPO-Porto), Porto, Portugal

**Keywords:** Canine mammary tumors, *ESR1* gene, SNP, Genetic profile

## Abstract

**Background:**

Estrogens are essential for the development and proper function of several hormone-dependent organs. There are, however, several lines of evidence associating estrogens with mammary carcinogenesis. A marked individual genetic variability concerning estrogens biosynthesis, metabolism and mechanism of action was recognized and associated with human breast cancer susceptibility, clinical features and progression. Although some genetic variations in canine *ESR1 gene* were reported, their influence in clinicopathological features and progression of canine mammary tumors has not been fully evaluated. This study aims to assess the influence of SNPs in *ESR1 gene* (rs397512133, rs397510462, rs851327560, rs397510612, rs852887655, rs852684753 and rs852398698) in canine mammary tumors characteristics and progression. A group of 155 non-neutered bitches with mammary tumors was included in the study. Follow-up information was assessed 24 months after surgery.

**Results:**

Genetic profiles associated with a later onset of mammary tumors and less aggressive clinicopathological features, namely smaller tumor size (≤ 3 cm) with extensive tubular differentiation and low canine-adapted prognostic index (vet-NPI), were identified in this study.

**Conclusions:**

Our data suggest that the *ESR1* genetic profile may help on the decision regarding the selection of individual tailored preventive measures against canine mammary tumors development, such as early neutering.

## Background

Estrogens are crucial for normal development and function of several organs and systems, namely the mammary gland. In women, estrogens play a pivotal role in the development of the mammary branching ductal-alveolar system in puberty, throughout the menstrual cycle and also during pregnancy [1–5].

Due to their pro-proliferative and anti-apoptotic affects, estrogens have also been implicated in human breast cancer development and progression [6–8]. Furthermore, some of the intermediate compounds derived from estrogens metabolism have a well-recognized genotoxic action [7, 9, 10]. Indeed, several conditions related to increased or prolonged exposure of the mammary tissue to estrogens, such as early menarche, late menopause, oral contraception or hormone replacement therapy, constitute well-known risk factors for human breast cancer [11].

There is a large body of evidence linking estrogens to mammary carcinogenesis, also in canine species. Most mammary tumors are reported in females and the few cases documented in males are related to estrogen-secretor testicular neoplasms [12, 13]. Besides, canine contraceptive hormonal therapy has long been associated to an increased risk of mammary tumors [14]. On the other hand, the protective effect of ovariectomy against the development of mammary neoplasia has been advocated for decades [15–17]. Moreover, levels of serum steroid hormones were reported to be higher in dogs with mammary carcinomas than in normal ones [18, 19].

Estrogens bind to estrogen receptors (ER) found in the normal canine mammary gland. Several studies reported changes in the expression pattern of ER in canine mammary gland in the course of neoplastic transformation and progression. An underexpression of ER has been documented in canine malignant mammary tumors, compared to benign neoplasms and to the normal mammary tissue [20–23]. This feature reinforces the importance of estrogens in canine mammary carcinogenesis. Furthermore, the decreased ER expression has been related to larger tumor size and lymph node metastasis, suggesting that ER status can be regarded as a marker with predictive and prognostic value in canine mammary tumors [21, 22].

Over the last decades, a considerable individual genetic variability concerning estrogens biosynthesis, metabolism and mechanism of action was recognized in humans. This individual genetic background is considered a significant contributor to breast cancer susceptibility, allowing the identification of subpopulations of women with higher breast cancer risk [24–27]. It has also been related to specific breast cancer clinical features, as well as to the clinical course of the disease [28–30]. In canine species data regarding the genetic profile related to estrogens and mammary tumor risk is not completely understood. Some *ESR1* genetic differences were described between different dog breeds known to be at high and at low risk of mammary tumor development; furthermore, an association between *ESR1* variation and the susceptibility to mammary tumors was described in a cohort of English Springer Spaniels [31, 32]. However, in a recent investigation, our group could not confirm a relationship between *ESR1* genetic profile and the risk of development of mammary tumors [33]. On the other hand, genetic variations in canine *COMT* gene (which encodes catechol-O-methyltransferase, an enzyme involved in estrogens metabolism through inactivation of carcinogenic catechol estrogens) has not been proved to influence susceptibility to canine mammary tumors. Nevertheless, *COMT* genetic variation has been linked to the age of onset of canine mammary carcinomas, to the development of high-grade mammary carcinomas, vascular invasion and recurrences [34–36]. To date, and to the best of the author’s knowledge, the influence of *ESR1* genetic profile in clinicopathological features and progression of canine mammary tumors has not been fully assessed.

The aim of this study is to investigate the association between seven single nucleotide polymorphisms (SNPs) in canine *ESR1* gene (rs397512133, rs397510462, rs851327560, rs397510612, rs852887655, rs852684753 and rs852398698) and clinicopathological features of canine mammary tumors and the clinical outcome of the disease.

## Results

One hundred and fifty-five non-neutered bitches were included in this study. The mean age of the whole population was 10.1 years-old (7–18 years-old): 9.8 years for dogs with benign tumors (*n* = 56; 36.1%) and 10.3 years for dogs with at least one malignant tumor (*n* = 99; 63.9%). Multiple tumours were diagnosed in 69.0% of the cases. Based on the Nottingham histological grading method 25/77 (32.5%) carcinomas were graded I, 36/77 (46.8%) were graded II and 16/77 (20.8%) were graded III. Vascular invasion and lymph node metastasis were observed in 14/96 (14.6%) and in 20/85 (23.5%) of cases, respectively. Two-year follow-up data was available for 88 dogs with malignant tumors. Of those, 44/88 (50%) were alive at the end of the follow-up period, while 21/88 (23.9%) died due to progression of the disease. Animals lost to follow-up (*n* = 16/88; 18.2%) and animals that died from causes not related to the mammary neoplasia (*n* = 7/88; 8%) were censored.

**Fig. 1:**
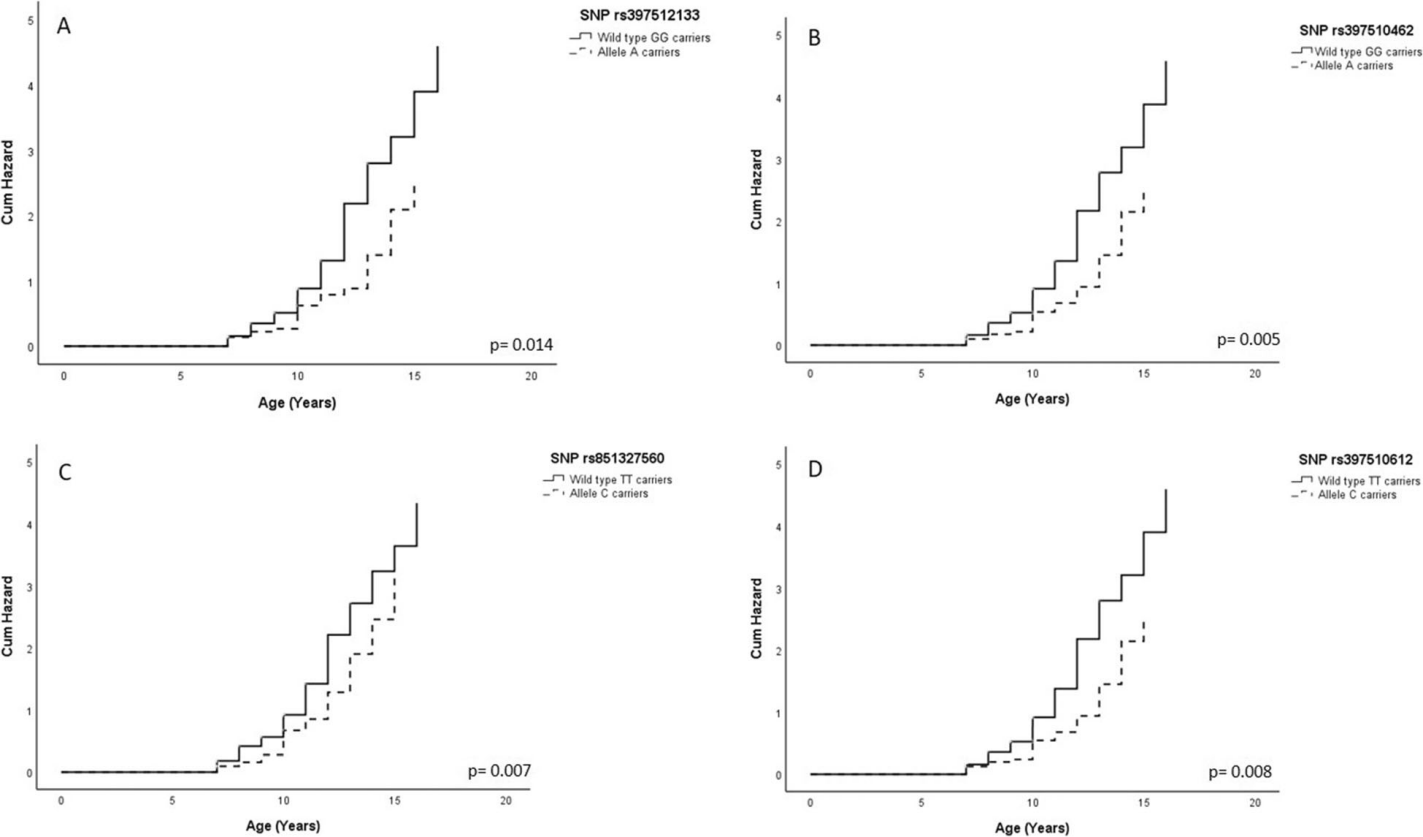
Association between the animal’s age at the time of the tumor diagnosis and SNPs of *ESR1* gene. A: SNP rs397512133; B: SNP rs397510462; C: SNP rs851327560 and D: SNP rs397510612

A significant association was found between the animal’s age at the time of the tumor diagnosis and SNPs rs397512133, rs397510462, rs851327560 and rs397510612. In fact, carriers of the variant allele for these SNPs developed mammary tumors later than wild type dogs (*p* = 0.014; *p* = 0.005; *p* = 0.007 and *p* = 0.008, respectively) (Fig. 1).

Table 1 presents results regarding the association between the studied SNPs and clinicopathological parameters, namely tumor number and size, histological classification (benign/malignant), mode of growth, NHG histological grade, NHG grading parameters (tubule formation, nuclear pleomorphism and mitotic index), NHG total score, vet-NPI, vascular invasion and lymph node metastases.

**Table 1:** Association between canine *ESR1* SNPs and clinicopathological features of canine mammary tumours

Single Nucleotide Polymorphisms (n = number of cases)																					
*Clinicopathological Variables*	rs397512133		*p*	rs397510462		*p*	rs851327560		*P*	rs397510612		*P*	rs852887655		*p*	rs852684753		*p*	rs852398698		*P*
	GG (n/%)	A Allele (n/%)		GG (n/%)	A allele (n/%)		TT (n/%)	C allele (n/%)		TT (n/%)	C allele (n/%)		GG (n/%)	A Allele (n/%)		Non DEL (n/%)	Del (n/%)		TTC (n/%)	Del/TTC (n/%)	
*Age*																					
< 10	79 (85.9	13 (14.1)		80 (87.0)	12 (13.0)		67 (72.8)	25 (27.2)		79 (85.9)	13 (14.1)		78 (84.8)	14 (15.2)		53 (58.2)	38 (41.8)		53 (57.6)	39 (42.4)	
≥ 10	46 (78.0)	13 (22.0)	NS	44 (74.6)	15 (25.4)	NS	35 (59.3)	24 (40.7)	NS	44 (74.6)	15 (25.4)	NS	49 (83.1)	10 (16.9)	NS	31 (53.4)	27 (46.6)	NS	31 (52.5)	28 (47.5)	NS
*Number of Tumors*																					
Single	40 (83.3)	8 (16.7)		41 (85.4)	7 (14.6)		36 (75.0)	12 (25.0)		40 (83.3)	8 (16.7)		43 (91.5)	4 (8.5)		26 (55.3)	21 (44.7)		26 (54.2)	22 (45.8)	
Multiple	88 (82.2)	19 (17.8)	NS	86 (80.4)	21 (19.6)	NS	69 (64.5)	38 (35.5)	NS	86 (80.4)	21 (19.6)	NS	87 (81.3)	20 (18.7)	NS	61 (57.5)	45 (42.5)	NS	61 (57.0)	46 (43.0)	NS
*Tumor Size* ^*α*^																					
≤ 3 cm	66 (78.6)	18 (21.4)		65 (77.4)	19 (22.6)		54 (64.3)	30 (35.7)		65 (77.4)	19 (22.6)		69 (83.1)	14 (16.9)		45 (53.6)	39 (46.4)		45 (53.6)	39 (46.4)	
> 3 cm	55 (91.7)	5 (8.3)	***0.034***	54 (90.0)	6 (10.0)	***0.049***	45 (75.0)	15 (25.0)	NS	54 (90.0)	6 (10.0)	***0.049***	51 (85.0)	9 (15.0)	NS	36 (62.1)	22 (37.9)	NS	36 (60.0)	24 (40.0)	NS
*Tumor Size (malignant)*																					
≤ 3 cm	35 (76.1)	11 (23.9)		34 (73.9)	12 (26.1)		26 (56.5)	20 (43.5)		34 (73.9)	12 (26.1)		35 (76.1)	11 (23.9)		26 (56.5)	20 (43.5)		26 (56.5)	20 (43.5)	
> 3 cm	46 (93.9)	3 (6.1)	***0.014***	45 (91.8)	4 (8.2)	***0.020***	39 (79.6)	10 (20.4)	***0.016***	45 (91.8)	4 (8.2)	***0.020***	43 (87.8)	6 (12.2)	NS	31 (64.6)	17 (35.4)	NS	31 (63.3)	18 (36.7)	NS
*Histological type*																					
*Benign*	43 (76.8)	13 (23.2)		44 (78.6)	12 (21.4)		37 (66.1)	19 (33.9)		43 (76.8)	13 (23.2)		49 (89.1)	6 (10.9)		28 (50.9)	27 (49.1)		28 (50.0)	28 (50.0)	
*Malignant*	85 (85.9)	14 (14.1)	NS	83 (83.8)	16 (16.2)	NS	68 (68.7)	31 (31.3)	NS	83 (83.8)	16 (16.2)	NS	81 (81.8)	18 (18.2)	NS	59 (60.2)	39 (39.8)	NS	59 (59.6)	40 (40.4)	NS
*Mode of growth*																					
*Expansive*	31 (81.6)	7 (18.4)		29 (76.3)	9 (23.7)		23 (60.5)	15 (39.5)		29 (76.3)	9 (23.7)		32 (84.2)	6 (15.8)		22 (59.5)	15 (40.5)		22 (57.9)	16 (42.1)	
*Infiltrative plus Invasive*	54 (88.5)	7 (11.5)	NS	54 (88.5)	7 (11.5)	NS	45 (73.8)	16 (26.2)	NS	54 (88.5)	7 (11.5)	NS	49 (80.3)	12 (19.7)	NS	37 (60.7)	24 (39.3)	NS	37 (60.7)	24 (39.3)	NS
*NHG Parameters*																					
*Tubule formation*																					
*Score 1 plus Score 2*	12 (70.6)	5 (29.4)		12 (70.6)	5 (29.4)		8 (47.1)	9 (52.9)		12 (70.6)	5 (29.4)		36 (73.5)	13 (26.5)		24 (49.0)	25 (51.0)		24 (49.0)	25 (51.0)	
*Score 2*	29 (90.6)	3 (9.4)	NS	29 (90.6)	3 (9.4)	NS	23 (71.9)	9 (28.1)	NS	29 (90.6)	3 (9.4)	NS	26 (92.9)	2 (7.1)	***0.039***	21 (77.8)	6 (22.2)	***0.014***	21 (75.0)	7 (25.0)	***0.026***
*Nuclear pleomorphism*																					
*Score 1 plus Score 2*	52 (85.2)	9 (14.8)		50 (82.0)	11 (18.0)		40 (65.6)	21 (34.4)		50 (82.0)	11 (18.0)		48 (78.7)	13 (21.3)		35 (58.3)	25 (41.7)		35 (57.4)	26 (42.6)	
*Score 2*	13 (81.3)	3 (18.8)	NS	13 (81.3)	3 (18.8)	NS	11 (68.8)	5 (31.3)	NS	13 (81.3)	3 (18.8)	NS	14 (87.5)	2 (12.5)	NS	10 (62.5)	6 (37.5)	NS	10 (62.5)	6 (37.5)	NS
*Mitotic count*																					
*Score 1 plus Score 2*	44 (83.0)	9 (17.0)		43 (81.1)	10 (18.9)		34 (64.2)	19 (35.8)		43 (81.1)	10 (18.9)		41 (77.4)	12 (22.6)		29 (55.8)	23 (44.2)		29 (54.7)	24 (45.3)	
*Score 3*	21 (87.5)	3 (12.5)	NS	20 (83.3)	4 (16.7)	NS	17 (70.8)	7 (29.2)	NS	20 (83.3)	4 (16.7)	NS	21 (87.5)	3 (12.5)	NS	16 (66.7)	8 (33.3)	NS	16 (66.7)	8 (33.3)	NS
*Total score*																					
*Score < 7*	40 (87.0)	6 (13.0)		39 (84.8)	7 (15.2)		31 (67.4)	15 (32.6)		39 (84.8)	7 (15.2)		35 (76.1)	11 (23.9)		25 (55.6)	20 (44.4)		25 (54.3)	21 (45.7)	
*Score ≥ 7*	25 (80.6)	6 (19.4)	NS	24 (77.4)	7 (22.6)	NS	20 (64.5)	11 (35.5)	NS	24 (77.4)	7 (22.6)	NS	27 (87.1)	4 (12.9)	NS	20 (64.5)	11 (35.3)	NS	20 (64.5)	11 (35.5)	NS
*NHG*																					
*Grade I plus Grade II*	50 (82.0)	11 (18.0)		49 (80.3)	12 (19.7)		38 (62.3)	23 (37.7)		49 (80.3)	12 (19.7)		47 (77.0)	14 (23.0)		33 (55.0)	27 (45.0)		33 (54.1)	28 (45.9)	
*Grade III*	15 (93.8)	1 (6.3)	NS	14 (87.5)	2 (12.5)	NS	13 (81.3)	3 (18.8)	NS	14 (87.5)	2 (12.5)	NS	15 (93.8)	1 (6.3)	NS	12 (75.0)	4 (25.0)	NS	12 (75.0)	4 (25.0)	NS
*Vet-NPI*																					
*≤ 4.0*	35 (77.8)	10 (22.2)		34 (75.6)	11 (24.4)		25 (55.6)	20 (44.4)		34 (75.6)	11 (24.4)		33 (73.3)	12 (26.7)		24 (54.5)	20 (45.5)		24 (53.3)	21 (46.7)	
*> 4.0*	29 (93.5)	2 (6.5)	NS	28 (90.3)	3 (9.7)	NS	25 (80.6)	6 (19.4)	***0.023***	28 (90.3)	3 (9.7)	NS	28 (90.3)	3 (9.7)	NS	20 (64.5)	11 (35.5)	NS	20 (64.5)	11 (35.5)	NS
*Vascular invasion*																					
*No*	68 (82.9)	14 (17.1)		66 (80.5)	16 (19.5)		53 (64.6)	29 (35.4)		66 (80.5)	16 (19.5)		66 (80.5)	16 (19,5)		48 (59.3)	33 (40.7)		48 (58.5)	34 (41.5)	
*Yes*	14 (100.0)	0 (0.0)	NS	14 (100.0)	0 (0.0)	NS	12 (85.7)	2 (14.3)	NS	14 (100.0)	0 (0.0)	NS	12 (85.7)	2 (14.3)	NS	9 (64.3)	5 (35.7)	NS	9 (64.3)	5 (35.7)	NS
*Lymph node metastases*																					
*No*	54 (83.1)	11 (16.9)		52 (80.0)	13 (20.0)		41 (63.1)	24 (36.9)		52 (80.0)	13 (20.0)		51 (78.5)	14 (21.5)		38 (59.4)	26 (40.6)		38 (58.5)	27 (41.5)	
*Yes*	19 (95.0)	1 (5.0)	NS	19 (95.0)	1 (5.0)	NS	17 (85.0)	3 (15.0)	NS	19 (95.0)	1 (5.0)	NS	18 (90.0)	2 (10.0)	NS	13 (65.0)	7 (35.0)	NS	13 (65.0)	7 (35.0)	NS

^α^ Including benign and malignant tumors

Legend: *NHG* Nottingham histological grade, *WHO* World Health Organization, *vet-NPI* Veterinary-adapted Nottingham Prognostic Index

Carriers of variant allele for SNPs rs397512133, rs397510462, rs851327560 and rs397510612 developed smaller size carcinomas (≤ 3 cm) than wild type animals (*p* = 0.014; *p* = 0.020; *p* = 0.016; p = 0.020, respectively). The NHG grading parameters (tubule formation, nuclear pleomorphism and mitotic counts) were evaluated and a statistically significant relationship was observed between SNPs rs852887655, rs852684753 and rs852398698 and tubule formation (*p* = 0.039; p = 0.014; *p* = 0.026, respectively). The majority of carcinomas scored 3 for tubule formation were found in wild type animals. In fact, only 7.1% (rs852887655), 22.2% (rs852684753) and 25.0% (rs852398698) of the carcinomas scored 3 for this parameter corresponded to variant allele carriers. Furthermore, a statistically significant association was found between rs851327560 and the vet-NPI (*p* = 0.023). Most cases of vet-NPI > 4 (80.6%) corresponded to wild type animals, while only 19.4% of those cases were observed in variant allele carriers.

No significant associations could be established between any of the SNPs considered and the number of tumors, histological classification (benign/malignant tumors) and pattern of tumor growth, NGH histological grade, nuclear pleomorphism, mitotic counts, vascular invasion and lymph node metastasis. Furthermore, none of the SNPs considered were related to OS.

## Discussion

In this study genetic variations of the canine *ESR1* were associated with the development of less aggressive canine mammary tumors.

Our results demonstrated that carriers of the variant allele for SNPs rs397512133, rs397510462, rs851327560 and rs397510612 tended to develop mammary neoplasia later in life than wild type dogs. This result finds parallel in data from a previous investigation of our group demonstrating that SNP in canine *COMT* gene were associated with the age of onset of mammary tumors [36]. According to that study, variant allele carriers for SNP rs853046495 (also known as SNP *COMT* G482A) presented a threefold likelihood of developing mammary tumors after 9 years of age, when compared to wild type animals. Furthermore, a significantly longer waiting time of onset of malignant disease was observed in variant allele carriers than in wild type animals.

The presence of the variant allele for SNPs rs397512133, rs397510462, rs851327560 and rs397510612 was also associated with the development of small size malignant tumors (≤ 3 cm). Tumor size has long been considered an important prognostic factor in canine mammary neoplasia, with tumors larger than 3 cm being associated with short survival times [37–40].

Our data also demonstrated that variant allele genotypes for SNPs rs852887655, rs852684753 and rs852398698 were associated with carcinomas with a high percentage of tubular arrangements, a feature related to well differentiated and low-malignancy neoplasias, and usually associated with good prognosis [41].

Despite the fact that none of the SNPs studied was related to NHG histological grade, carriers of the variant allele for rs851327560 were significantly associated with low vet-NPI (≤4). This recently described index combines well-recognized prognostic factors for canine mammary tumors, namely tumor size, NHG histological grade and vascular/lymph node invasion. Previous studies from our group demonstrated that this canine-adapted index is associated with disease free-interval and OS in bitches [42, 43].

Taken together, our results allow the identification of a subgroup of dogs that tend to develop mammary tumors at older ages and with less aggressive clinicopathological features (small size tumors, with extensive tubular differentiation and low vet-NPI). Based on these findings, we hypothesize that carriers of the variant allele for *ESR1* SNPs (rs397512133, rs397510462, rs851327560, rs397510612, rs852887655, rs852684753 and rs852398698) may possess receptors less sensitive to estrogen binding, resulting in a mammary tissue less responsive to the hormone, thus being more protected from its carcinogenic action. It is conceivable that in those dogs, a longer period of exposure to estrogens would be required to achieve carcinogenic levels, which could explain the development of tumors in older age. Despite the fact that SNPs assessed in this study are synonymous or intronic, and do not involve amino acid changes, they can induce alternative splicing, promote changes in RNA stability or structure, interfere with the speed and accuracy of transcription and/or translation and disturb protein folding [44–47]. Even if there is no change in protein production, as it happens in non-synonymous, the allegedly subtle characteristics produced by synonymous and intronic SNPs may explain the diversity of individual reaction to hormonal stimulus exhibited by different animals.

There are several epidemiological, biochemical and toxicological lines of evidence associating estrogens with mammary carcinogenesis [9, 10, 48, 49]. However, estrogens are also essential for the development and proper function of several hormone-dependent organs [50–52]. In fact, there are various detrimental effects related to neutering, such as urinary incontinence, musculoskeletal disorders and development of different types of neoplasia [53–64]. Besides, several sources of bias were identified in previous studies that link neutering to a decreased risk of mammary neoplasia. A more recent revision of the data previously available was performed, demonstrating that the scientific support for this evidence is weak [61]. In this vein, a growing debate on whether and when spaying should be recommended has emerged. Several researchers have questioned the value of early spaying, taking into account the secondary effects of this procedure in the animal’s health and quality of life in the medium and long term [54, 60, 62]. In fact, the relationship between gonadectomy, cancer development, overall lifespan and cancer-related death is complex. Besides, biological and molecular mechanisms involved in this process are poorly understood. The conflicting data obtained from different studies may be related to the individual genetic profile of the animal, namely to SNPs.

It is important to identify subsets of animals with increased risk or more susceptible to the development of aggressive mammary tumors that, in spite of the medium-long term secondary effects resulting from neutering, could benefit from that intervention. Similarly, recognition of the animals in which the deleterious consequences from neutering overlap their protective effect against the development of mammary neoplasia is of uttermost importance, avoiding unnecessary surgical interventions. In this sense, further research must be conducted considering other genetic variants and involving a larger number of animals, in order to assess the importance of the individual genetic profile in gonadectomy effects.

## Conclusions

Our data suggest that canine *ESR1* genetic profile may constitute a rational basis for the evaluation of the cost-benefit ratio related to early spaying. However, additional broad investigation is needed to expand these findings and to clarify the relevance of the genetic profile, assisting in the selection of the animals that could benefit from neutering as an individualized preventive strategy against the development of canine mammary neoplasia.

## Methods

The study was conducted involving 155 non neutered bitches with histologically confirmed mammary tumors collected from the Veterinary Pathology Laboratory (ICBAS-University of Porto). All animals were treated with radical unilateral and/or partial mastectomy. Owners provided consent for surgery with curative intents as well as for the use of the material for research purposes. This protocol was approved by the Ethics Committee of the Institute of Biomedical Sciences Abel Salazar, University of Porto (P151/2016).

After surgery, mammary specimens were immediately fixed in a 10% buffered formalin solution and routinely processed for histopathological examination. For each case, clinicopathological features including age at the time of the diagnosis, tumor number and size (corresponding to the largest diameter measured by a pathologist (ACS) during trimming) were recorded. The histological diagnosis was established by consensus of 3 pathologists (ACS, MS and PDP) in a multi-head microscope, using the criteria of the World Health Organization for the classification of mammary tumors of dogs and cats [65]. Each malignant tumor was assessed for the mode of growth and the presence of vascular invasion and regional lymph node metastasis, as previously described [43]. In the subgroup of animals with multiple malignant tumors, a reference lesion was assigned for the statistical study, according to criteria previously reported [43]. Histological grading was performed based on the Nottingham histological grading method -NHG [66]. A veterinary adaptation of the human Nottingham Prognostic Index (vet-NPI) was also computed, as previously reported [42].

Follow-up data was obtained by consulting the medical records and by contact with the referring veterinarian. Disease-specific overall survival (OS) was calculated from the time of diagnosis to the date of the animal’s death/euthanasia due to the neoplastic disease. Animals that died or were euthanized for unrelated causes and those that were lost to follow-up were censored, respectively, at the time of death and at the data of their last clinical examination. Euthanasia was performed only in terminal stage of the disease. Necropsy examination was performed upon the owner consent.

Genomic DNA was extracted from peripheral blood samples (obtained by standard venipuncture) using High Pure PCR Template preparation kit (Roche). The DNA quality was evaluated by measuring the optical density and the quantity was assessed employing the NanoDrop 1000 Spectrophotometer. SNP genotyping was performed using MassARRAY iPLEX Gold Technology at the *Unidade de Genómica/Serviço de Genotipagem do Instituto Gulbenkian de Ciência.* This technology for SNP genotyping consists of an initial PCR reaction, followed by multiplexed primer extension (using mass-modified dideoxynucleotide terminators of an oligonucleotide primer) which anneals immediately upstream of the polymorphic site of interest. Additionally, the MALDI-TOF (matrix assisted laser desorption/ionization - time of flight) mass spectrometry allows the recognition of the SNP allele by the different mass of the extended primer [67, 68].

**Fig. 2:**
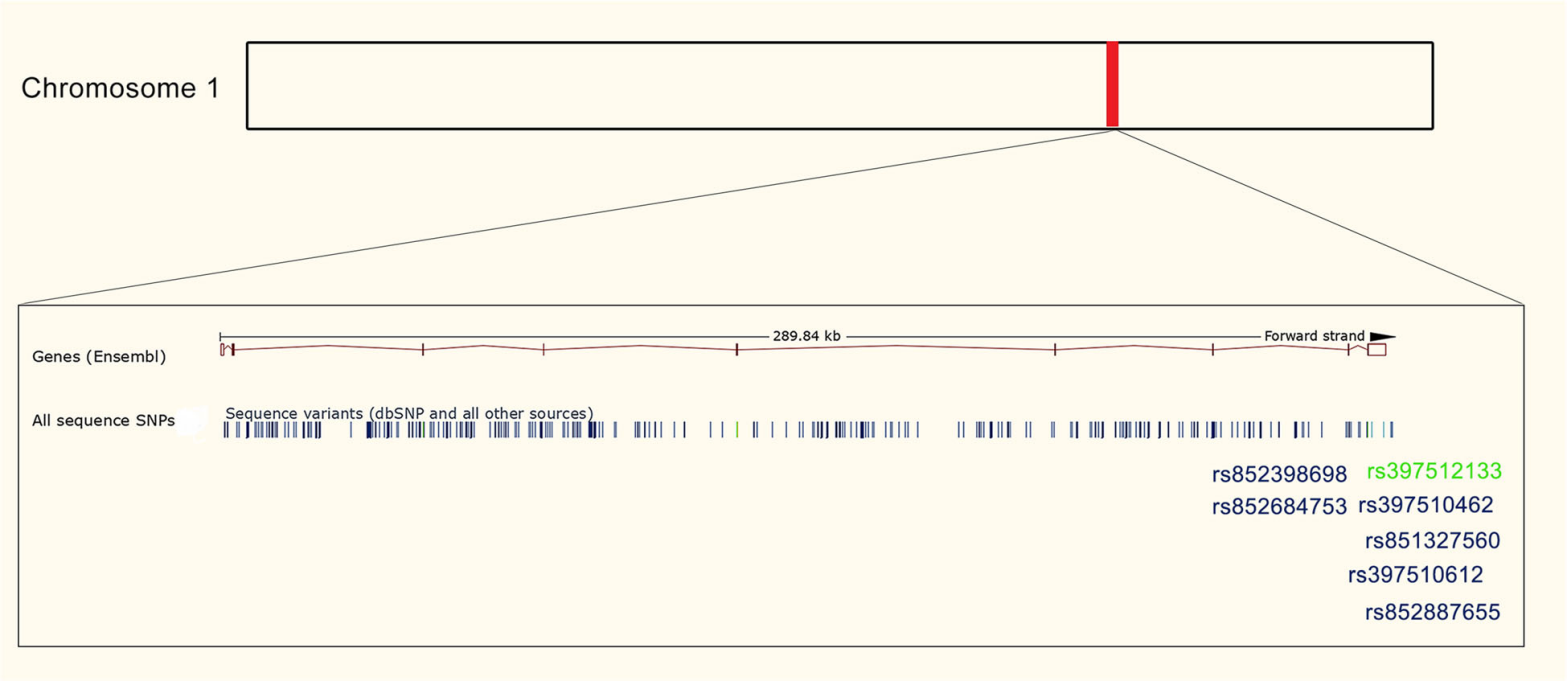
Schematic illustration of the selected SNPs of *ESR1* gene located in the chromosome 1. The selected SNPs are represented on the illustration by their cluster ID numbers in the public SNP database. Coding regions are shown as a box on a horizontal bar whereas the horizontal bar represents the noncoding region of the illustrated fragment. Green: synonymous SNP; blue: intronic SNPs

Seven canine *ESR1* (chromosome 1) SNPs were assessed as referred in Table 2 and Fig. 2. Statistical analysis of data was performed using the computer software SPSS for Windows (version 25). Chi-square analysis (or Fisher’s exact test, when appropriated) was used to evaluate the significance of the relationship between *ESR1* SNPs and the categorical variables. Cumulative risk curves were computed using Kaplan-Meier product-limit estimates method, with Log-rank (Mantel-Cox) tests being used to estimate the differences in risk of canine mammary tumors development according to each genetic profile. A 5% level was considered to define statistical significance.

**Table 2:** SNPs assessed in this study

SNP	Type	Location	Change	Amino acid
rs397512133	Synonymous	Exon 8	G- > A: CTG-CTA	Leucine
rs397510462	Intronic	42.32 Mb to 42.37 Mb	G- > A	–
rs851327560	Intronic	42.32 Mb to 42.37 Mb	T- > C	–
rs397510612	Intronic	42.32 Mb to 42.37 Mb	T- > C	–
rs852887655	Intronic	42.32 Mb to 42.37 Mb	G- > A	–
rs852684753	Intronic	42.32 Mb to 42.37 Mb	TTTTC/−	–
rs852398698	Intronic	42.32 Mb to 42.37 Mb	TTC/−	–

## Data Availability

The datasets used and analyzed for this study are available from the corresponding author on reasonable request.

## Abbreviations

COMT: Catechol-O-methyltransferase
ER: Estrogen receptors
ESR1: Estrogen receptor 1
MALDI-TOF: Matrix assisted laser desorption/ionization - time of flight
NHG: Nottingham histologic grade
OS: Overall survival
SNP: Single nucleotide polymorphism
vet-NPI: Veterinary adapted Nottingham Prognostic Index
